# Communicating uncertainty in seasonal and interannual climate forecasts in Europe

**DOI:** 10.1098/rsta.2014.0454

**Published:** 2015-11-28

**Authors:** Andrea L. Taylor, Suraje Dessai, Wändi Bruine de Bruin

**Affiliations:** 1 Sustainability Research Institute and ESRC Centre for Climate Change Economics and Policy, School of Earth and Environment, University of Leeds, Leeds, UK; 2 Centre for Decision Research, Leeds University Business School, University of Leeds, Leeds, UK; 3 Department of Engineering and Public Policy, Carnegie Mellon University, Pittsburgh, PA, USA

**Keywords:** seasonal climate forecast, decadal prediction, communicating uncertainty, risk communication, Europe

## Abstract

Across Europe, organizations in different sectors are sensitive to climate variability and change, at a range of temporal scales from the seasonal to the interannual to the multi-decadal. Climate forecast providers face the challenge of communicating the uncertainty inherent in these forecasts to these decision-makers in a way that is transparent, understandable and does not lead to a false sense of certainty. This article reports the findings of a user-needs survey, conducted with 50 representatives of organizations in Europe from a variety of sectors (e.g. water management, forestry, energy, tourism, health) interested in seasonal and interannual climate forecasts. We find that while many participating organizations perform their own ‘in house’ risk analysis most require some form of processing and interpretation by forecast providers. However, we also find that while users tend to perceive seasonal and interannual forecasts to be useful, they often find them difficult to understand, highlighting the need for communication formats suitable for both expert and non-expert users. In addition, our results show that people tend to prefer familiar formats for receiving information about uncertainty. The implications of these findings for both the providers and users of climate information are discussed.

## 1. Introduction

Across Europe, decision-makers in sectors as diverse as water management, agriculture, energy, health, transportation and tourism face the challenge of adapting to climate variability and change. Potential increases in the intensity and frequency of extreme events such as flooding, heatwaves and water shortages could lead to property damage, losses in profit and even human casualties. Seasonal and interannual climate forecasts therefore have potentially high value for these organizations [1,2]. Decision-makers have traditionally relied on historical data (i.e. climatology) to predict future seasonal and annual averages. However, improved climate forecasts now outperform historical averages for forecasts of European winters [3]. If the quality of seasonal forecasts for Europe continues to improve, their use is likely to increase. Indeed, future developments in interannual climate modelling (also known as decadal prediction in the literature) may mean that forecasts for up to 10 years in the future become more widely available [4]. If so, decision-makers will need communications that explain the uncertainty associated with these forecasts.

The question of how uncertainty should be communicated is one faced by scientists in many fields [5]. If uncertainty is not adequately communicated, then it can cause recipients to experience a false sense of certainty [6,7], maladaptive decision-making [8,9] and, if discovered, diminished trust in communicators [9,10]. However, research in the behavioural decision sciences shows that people—even experts such as medical doctors—can struggle to understand probabilistic information [11,12]. Additionally, recipients may discount information that is perceived as uncertain [13,14]. In contexts such as climate risk management, where greater uncertainty is often associated with greater expected damages [15], this could lead to costly failures to act [16].

This article focuses on the communication of uncertainty in seasonal and interannual climate forecasts. As with other model-based prediction systems, uncertainty in climate forecasts comes from both the probabilistic nature of the forecast, and the fact that the models used to make these forecasts do not have perfect reliability [17]. Hence, users must be aware of both types of uncertainty, if they are to make informed decisions about whether or not to use seasonal or interannual forecasts, and—if so—how to use them in their decision-making. However, the complex ways in which climate scientists represent uncertainty may not be well understood by users, or provide them with the information they need to make decisions. To identify strategies for effectively communicating uncertainty to users in climate-sensitive organizations, it is important to first understand users’ information needs, preferences and level of understanding [18,19].

In §2, we discuss some of the challenges faced by providers of climate forecasts, while referencing the risk perception literature. Section 3 describes the survey we conducted to explore user needs and preferences regarding uncertainty in seasonal and interannual climate forecasts. In §§4–6, we present key findings regarding organizational approach to uncertainty, perceptions of accessibility, understandability and usefulness, as well as user preferences for receiving uncertain information. Section 7 discusses the findings and their implications for providers and users of climate information.

## 2. Challenges in communicating uncertainty in the context of seasonal and interannual forecasts

When predicting future climate, uncertainty arises from multiple sources. In the large and multi-disciplinary literature on uncertainty, a distinction is typically drawn between first-order and second-order uncertainty [17]. First-order uncertainty refers to the likelihood of an event happening according to a particular forecast and is also referred to as aleatory uncertainty, probability or risk. Second-order uncertainty, also known as epistemic uncertainty, Knightian uncertainty or ambiguity, refers to ‘uncertainty about the uncertainty’. Among other things, second-order uncertainty may result from whether the model used to make the forecast includes all of the right inputs, whether the relationship between these inputs is accurately captured and whether inputs are correctly measured [17]. Seasonal and interannual forecasts contain both forms of uncertainty, because of the chaotic nature of the climate system and our imperfect knowledge of the system. To represent first-order uncertainty, multiple simulations are used to produce a probability distribution [20]. However, as forecasts cannot capture all of the factors influencing the climate, second-order ‘epistemic’ uncertainty also exists. This second-order uncertainty is typically represented as a measure of reliability (how well predictions have matched observations) or skill (how well predictions have performed relative to historical averages). To effectively communicate these types of uncertainty, forecast providers must contend with potential mismatches between providers and users in their information preferences and understanding, variations in tolerance for uncertainty, as well as a dearth of evidence-based recommendations as to how best to communicate uncertainty in this context.

### (a) Mismatch between providers and users’ understanding of uncertainty and preference for receiving uncertain information

When communicating climate information, forecasters face trade-offs between richness (level of detail provided), robustness (appropriate reflection of reliability and limitations), and the ease with which information can be understood and used [21]. Even if richly detailed forecasts can be produced reliably, users may not understand them. By definition, those producing seasonal and interannual forecasts are experts in their field. Users of climate forecasts may however include individuals who are less familiar with statistics. Should the use of seasonal forecasts become more widespread, the proportion of such users may grow. Research examining mathematical ability (numeracy) [22] and graph comprehension (graph literacy) [23] shows that even educated members of the public may struggle to understand probabilistic and graphical information [12]. If this is not taken into account by communicators, then users may disregard the information provided, or misinterpret it [24]. This does not mean that communicating probabilistic forecasts to non-experts poses an insurmountable challenge. Recent studies of the interpretation of probabilistic weather forecasts have demonstrated that non-experts can use information about uncertainty in decision-making when its presentation format is compatible with decision task and the recipients’ cognitive processes (e.g. when it is made clear that a critical threshold is inside or outside an interval forecast, when the probability of a critical threshold being crossed is made salient) [25–27]. It does however mean that formats must be tested to ensure that information is interpreted as intended. Risk communication specialists therefore stress the importance of identifying where non-experts’ understanding of scientific information differs from that of experts [18,19]. For instance, a probabilistic temperature forecast that visually depicts the upper and lower bounds of a confidence interval may be misconstrued as a deterministic forecast for high and low daily temperatures [27]. Additionally, people often do not fully understand what the probabilities in weather forecasts refer to [28–30], and misinterpret a 70% chance of rain tomorrow as meaning that ‘it will rain for 70% of the day tomorrow’ rather than ‘on 70% of days like tomorrow it rains’. These ‘reference class’ errors may not always be a barrier to making decisions [31], such as carrying an umbrella where there is a high likelihood of rain. However, as the operational and strategic choices made within organizations can have far-reaching consequences for their success, a better understanding of probabilities may be desired, to promote more informed decisions about what actions to take in response to climate forecasts [32].

We should note that non-expert users may ignore forecasts that they perceive as too complex [33]. People are often less motivated to engage with difficult information, leading them to focus on cues that are easier to understand but potentially less relevant [34]. However, where users have experience of working with information about climate and climate impacts more complex, richly detailed representations may actually be preferred if they are perceived to provide relevant information about the forecast [35]. Research on hurricane forecasting [36] and long-term climate projections [35] suggests that people pay more attention to familiar communication formats. Unfortunately, formats that are familiar may not necessarily be the best understood [37–39]. It is therefore important to ascertain what user preferences are with respect to receiving uncertain information, so that these may then be tested for actual understanding.

Even among users with high technical and statistical expertise, forecasts may not always meet user requirements. For instance, where users are concerned with low-probability high-impact events and the available forecast information highlights only the likelihood of being above or below average, then it may be insufficient. Likewise, some users may wish to receive information in a way that provides clear Act/Don’t Act signals for action [40], and may find information formats that do not facilitate this difficult to use in decision-making. Hence, characterizing how users want to use uncertain information in their decision-making is important.

### (b) Tolerance for uncertainty

Behavioural decision research shows that people frequently demonstrate what has been termed ‘ambiguity aversion’, or a reluctance to select alternatives for which probabilities are not precisely represented as single point estimates [41]. However, findings are inconsistent. Some studies have reported that people prefer point estimates over ranges [13] and that wider confidence intervals may be perceived as less trustworthy [42,43]. However, others have found the reverse [44]. Likewise, studies of probabilistic weather forecasting suggest that ranges can be appropriately used to enhance the quality of judgement and decision-making by non-experts [25–27,45,46]. Indeed, people often infer uncertainty when presented with deterministic weather forecasts [29,47,48]. Because familiar presentation formats tend to be preferred [35,36], it is possible that the widespread availability of probabilistic weather forecasts has led to a greater tolerance for uncertainty in weather forecasts than for less familiar contexts, such as longer term climate forecasts. However, recent work examining the presentation of uncertainty in climate change projections has shown that tolerance for uncertainty may also depend on the type of uncertainty that is presented. That is, people were more supportive of climate change mitigation when they were presented with uncertainty about ‘time until impact occurs’ rather than ‘probability of impact occurring by a particular year’, despite the information being objectively identical [49]. It is, of course, possible to develop communications that take into account users’ risk appetite when producing cues or recommendations for action [50]. However, if this renders the presence of uncertainty less salient, then users may develop a false sense of certainty, which may lead to decision-making that is not in keeping with actual risk preference [6].

### (c) A dearth of recommendations

One overarching problem when attempting to address the issues outlined above is that there is, at present, a lack of empirically supported recommendations for presenting uncertainty in climate forecasts at seasonal and interannual time scales. The risk communication literature has introduced various formats for communicating uncertainty, but empirical support for their understandability and usefulness is still lacking [5]. This poses a considerable challenge for forecast providers seeking to communicate climate information to diverse audiences. Indeed, the difficulties inherent in producing a framework for representing uncertainty is highlighted by the response to the Intergovernmental Panel on Climate Change’s guidelines on consistent treatment of uncertainties [51]. One goal of these guidelines, which have separate scales for ‘likelihood’ and ‘confidence’ (level of expert agreement and amount of evidence), was to provide greater transparency about uncertainty in climate projections; however, they have been criticized for being overly complex [52] and open to inconsistent interpretation [53]. While the sources of uncertainty in long-term climate projections are not identical to those in seasonal and interannual forecasts, the challenge of finding ways to represent first- and second-order uncertainty is similar.

## 3. Survey design and methodology

### (a) Objectives

As a first step towards identifying effective strategies for communicating uncertainty in the context of seasonal and interannual climate forecasts, we conducted a survey that aimed to understand how participants’ organizations approach uncertain information in their decision-making (§4), how accessible, understandable and useful seasonal and interannual forecasts are perceived to be (§5), and what participants’ preferences are with respect to receiving information about uncertainty (§6).

### (b) Participants

Our survey was conducted as part of the EUPORIAS (European Provision of Regional Impact Assessments on Seasonal and Decadal Timescales) Project, which has been undertaken to develop the provision of climate services at seasonal and interannual time scales across Europe in a way that meets user needs [1]. Participants were recruited via e-mail from a pool of EUPORIAS stakeholders, and organizations who had expressed an interest in seasonal or interannual forecasts, even if they were not currently using them.

Between October 2013 and December 2013, 50 participants responded to an online survey. A total of 44 provided complete responses. A diverse range of sectors were represented, including water management, forestry, energy, health, tourism, agriculture, transportation, emergency services, climate consultancy and finance (see the electronic supplementary material for sectoral breakdown). As a whole, the sample was highly educated, with 32 out of 41 participants specifying their highest level of academic qualification at the postgraduate or doctoral level, and the remainder choosing not to answer this question.

### (c) Survey design and implementation

The survey was implemented using Qualtrics, a tool for online survey design. Key questions focused on organizational approach to uncertainty; understandability and usefulness of climate information; and preferences with respect to receiving information about uncertainty. These measures are detailed in §§4–6. The full survey can be found in the electronic supplementary material.

## 4. Organizational approach to uncertainty

Organizations may vary in their tolerance for uncertainty, their capacity for processing complex probabilistic information and the types of uncertain information they are interested in. To get a sense of how the organizations represented in our sample approached uncertainty, we asked participants to rate their agreement with a series of statements about (i) information processing and interpretation within their organization, (ii) focus on extreme versus most likely events, and (iii) tolerance for uncertainty.

### (a) Measures

**Figure 1.**
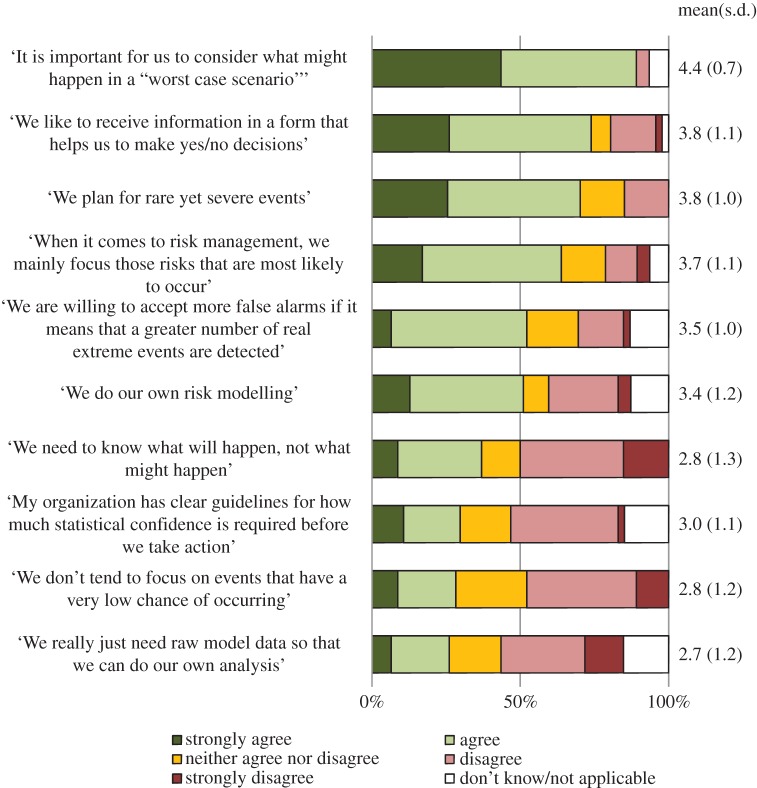
Level of agreement with each statement regarding organizational approach to uncertainty and decision-making (*n*=46–47).

Participants were asked to rate their agreement with the 10 statements in figure 1 on a scale of 1 (strongly disagree) to 5 (strongly agree), with an additional option to answer ‘Don’t Know/Not Applicable’. In the statistical analysis of these items, ‘Don’t Know/Not Applicable’ responses have been coded as missing data. Figure 1 shows the wording of each question, as well as the rate of agreement.

### (b) Information processing and interpretation

As seen in figure 1, a majority of participants agreed that their organization liked to receive information in a format that facilitated yes/no decision-making. However, when it came to questions regarding ‘in house’ data processing relatively few agreed that their organization had clear guidelines as to how much statistical confidence was needed before taking action, or that their organization only required raw climate and weather data. Just over half agreed that their organization did some form of risk modelling. Hence, we find that while a sizeable proportion of the organizations represented within our sample do perform some form of ‘in house’ data modelling, the majority require the providers of uncertain climate and weather information to perform at least some processing and interpretation.

### (c) Focus on extreme versus most likely events

Most participants agreed that their organization planned for ‘rare yet severe events’ and indicated that when it came to climate and weather it was important for them to consider ‘what might happen in a “worst case scenario” as well as what is most likely to happen’, with responses to these items being positively correlated (*n*=43, *ρ*=0.35, *p*=0.02). Meanwhile, comparatively few agreed that their organization *did not* tend to focus on ‘events that have a very low chance of occurring’, with responses to this question being negatively correlated with planning for rare yet severe events (*n*=46, *ρ*=−0.32, *p*=0.03), and concern with worst case scenarios (*n*=43, *ρ*=−0.36, *p*=0.02). However, despite this evident concern with low-likelihood high-impact events, a majority of participants agreed that their organization tended to focus on ‘those risks that are most likely to occur’. Agreement with this statement was not significantly associated with planning for rare yet severe events (*n*=44,*ρ*=−0.22, *p*=0.16), considering worst case scenarios (*n*=43, *ρ*=0.25, *p*=0.12), or refraining from focusing on low-probability events (*n*=40, *ρ*=0.08, *p*=0.60). Hence, while a majority organizations within the sample are primarily concerned with those events that are ‘most likely’ this does not preclude concern with low-likelihood high-impact events.

### (d) Tolerance for uncertainty and its relationship with information preference

Tolerance for uncertainty was relatively high among the sample, with more participants disagreeing than agreeing with the statement: ‘We need to know what will happen not what might happen’. Tolerance for false alarms was also relatively high, with just over half of participants agreeing that: ‘When it comes to predicting extreme weather events we are willing to accept more false alarms if it means that a greater number of real extreme events are detected in advance’. Interestingly however, no association was found between responses to these items (*n*=40, *ρ*=0.01, *p*=0.94).

We found that agreeing with ‘We need to know what will happen not what might happen’ was associated with greater preference for receiving information that facilitates yes/no decision-making (*n*=45, *ρ*=0.51, *p*<0.001). However, it was not associated with preference for receiving raw data (*n*=39, *ρ*=−0.08, *p*=0.65). We therefore find that tolerance for uncertainty among the sample is generally high, but that lower tolerance for uncertainty tends to correspond with a stronger preference for formats that provide recommendations for action.

## 5. Accessibility, understandability and usefulness

In working towards developing effective and understandable methods of communicating uncertainty in seasonal and interannual climate forecasts, it is important to establish how easy to access and interpret users perceive this information to be. In this section of the survey, we therefore sought to assess (i) how easy to access, easy to understand and useful current users perceived forecast information to be and (ii) how perceptions of seasonal and interannual climate forecasts compared to perceptions of weather forecasts.

### (a) Measures

Participants were first asked whether their organization currently received climate information for up to one month in the future (weather time scale) (*n*=45), from one month to 1 year in the future (seasonal time scale) (*n*=35), and from 1 to 10 years in the future (interannual time scale) (*n*=18). For each type of information currently received, they were then asked to rate how: easy to access or find, easy to understand and useful they perceived it to be on a scale of 1 (not at all) to 5 (very much so). An additional don’t know response was also available. Where don’t know was selected responses were excluded from the statistical analysis of these data.

### (b) How easy to access, easy to understand and useful do current users perceive forecast information to be, and does this differ between types of forecasts?

At each time scale, forecasts were perceived to be more useful than they were accessible or understandable, though this difference was more pronounced for seasonal and interannual forecasts than weather forecasts (table 1). Ratings of accessibility and understandability were strongly correlated with one another, but not with perceived usefulness. This pattern was observed for weather, seasonal and interannual forecasts. Hence, information that people perceive as useful is not necessarily information that they can easily access and understand.

**Table 1.** Mean ratings of ease of access, ease of understanding and usefulness of forecasts at different time scales (weather, seasonal and interannual), with intercorrelations between ease of access, ease of understanding and usefulness (Spearman’s *ρ*). Participants who indicated that their organization received a particular forecast but responded with don’t know when asked to rate ease of access, ease of understanding or usefulness have been excluded from calculations and analyses involving forecasts at that time scale.

	weather forecasts (*n*=41)	seasonal forecast (*n*=28)	interannual forecast (*n*=15)
	mean (s.d.)	mean (s.d.)	mean (s.d.)
easy to access	3.7 (1.1)^a^	2.5 (1.2)^*b*^	1.9 (0.8)
easy to understand	3.8 (1.0)^a^	2.8 (1.2)^b^	2.4 (0.9)
useful	4.6 (0.8)	4.3 (1.0)^b^	3.8 (0.8)

	*ρ*	*ρ*	*ρ*
access and understanding	0.62***	0.64***	0.74***
access and usefulness	0.23	0.09	0.28
understanding and usefulness	0.30^#^	0.05	0.32

Significant at *p≤0.05, **p≤0.01, ***p≤0.001; marginally significant at ^#^*p*≤0.10.

^a^Weather forecasts perceived as significantly easier to access (*n*=28, *z*=3.25, *p*≤0.001) and easier to understand (*n*=28, *z*=3.47, *p*≤0.001) than seasonal forecasts, but not more useful (*n*=28, *z*=1.35, *p*=0.18).

^b^Seasonal forecasts perceived as significantly easier to access (*n*=12, *z*=2.24, *p*=0.03), easier to understand (*n*=12, *z*=2.12, *p*=0.03) and more useful (*n*=12, *z*=2.65, *p*=0.008) than interannual forecasts.

As can be seen in table 1, mean ratings of accessibility, understandability and usefulness decreased as forecast lead time increased; being highest for weather forecasts and lowest for interannual forecasts. Additionally, weather forecasts were perceived to be significantly easier to access and understand than seasonal forecasts, but were not perceived as significantly more useful. Seasonal forecasts meanwhile were perceived to be significantly easier to access, easier to understand and more useful than interannual forecasts (table 1, footnote). As weather forecasts are more prevalent than seasonal or interannual forecasts, differences in perceived accessibility would seem to be in keeping with actual availability. However, the lower perceived understandability of seasonal and interannual forecasts is more concerning, as this indicates that the forecast information that is available does not tend to be user-friendly.

## 6. User preferences and information currently received

We also examined participants’ preferences with respect to the presentation of uncertainty in climate forecasts, and whether this corresponded with existing familiarity with these formats and comfort with statistics. We were interested in identifying what types of information about uncertainty current users of seasonal and interannual forecasts receive, and what they were not receiving that they would like to receive. As previously noted, preference for particular information formats is not always associated with better objective understanding [37]. However, as users may be more reluctant to use formats that are not in keeping with their preferences, it is important to understand what these are. We therefore sought to address the following: (i) Which methods of representing probability do participants prefer? (ii) To what extent is preference associated with familiarity and statistical comfort? (iii) What type of information about uncertainty in seasonal and interannual forecasts are users currently receiving, and what else would they like to receive?

### (a) Measures and visualizations

**Figure 2.**
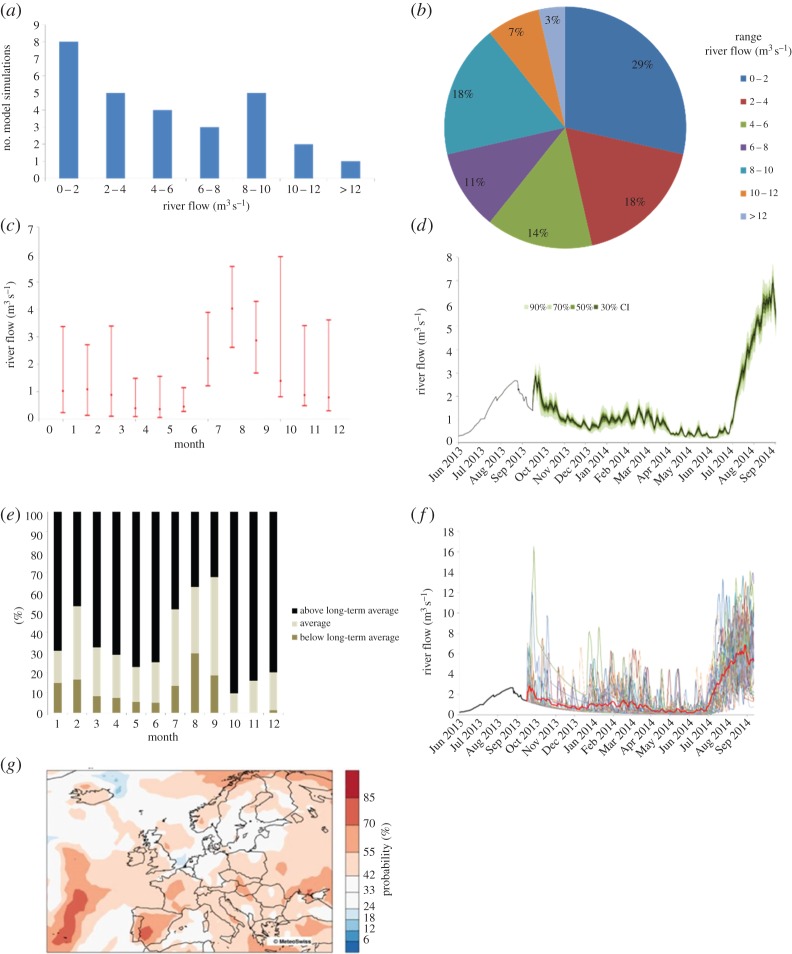
Visualizations rated for preference and familiarity. (*a*) Bar graph showing forecast distribution. (*b*) Pie chart. (*c*) Error bars. (*d*) Fan chart. (*e*) Tercile bar graph. (*f*) Spaghetti plot. (*g*) Map. (*a*–*f*) A hypothetical seasonal river flow forecast. (*g*) A hypothetical forecast for likelihood of above-average seasonal temperatures.

Seven visualizations depicting first-order uncertainty in a seasonal forecast were presented to participants (figure 2; but for full-sized visualizations and full captions, see electronic supplementary material). Six of these were graphs depicting a hypothetical seasonal river flow forecast: bar graph showing forecast distribution; pie chart; error bar; fan chart; tercile bar graph; and spaghetti plot (i.e. showing the likelihood of above average, average or below average conditions). One visualization was a map of Europe depicting a hypothetical forecast for likelihood of above-average seasonal temperatures. These formats were chosen as they are relatively common ways of representing probability [5]. All participants saw the visualization formats in the same order. Our sample size was not large enough to support counterbalancing of presentation order. As a result, responses may have been affected by fatigue or direction-of-comparison effects [54,55]. Ideally, studies comparing responses to different visualizations should use a between-groups design (i.e. randomly assign each participant a single visualization), or systematically counterbalance the order of presentation in a within-group design (i.e. assign different groups of participants to consider visualizations in a different order).

For each visualization, participants rated their agreement with five statements to indicate their preference (e.g. ‘This type of graph is useful’) on a scale of 1 (strongly disagree) to 5 (strongly agree). A preference score for each visualization was then calculated by taking the mean of these ratings. Participants also indicated their familiarity, by indicating their agreement (1=strongly disagree, 5=strongly agree) with the statement ‘I use graphs like this in my work’. Finally, they rated their level of statistical comfort on a three-point ordinal scale, with higher score representing greater comfort with more complex statistics: 1=‘I am not comfortable using statistics or numerical information’ or ‘I am comfortable using basic statistics and numerical information (e.g. means, percentages, frequency counts)’; 2=‘I am comfortable using more complex statistics and numerical information (e.g. confidence levels, probability distributions)’ or ‘I am comfortable using standard statistical tests (e.g. correlations, *t*-tests)’; 3=‘I am comfortable using more advanced statistical techniques (e.g. Monte Carlo simulations, mathematical modelling)’.

Those participants who reported that their organization received seasonal or interannual forecasts (*n*=32) were asked to indicate whether their organization currently received the following forms of information: ‘Ranges of values’, ‘Confidence intervals’, ‘Verbal descriptions of likelihood’, ‘Raw data’, ‘Probability distributions’, ‘Information about possible sources of error’, ‘Information about how well earlier forecasts have matched observed climate’, ‘Indicators of signal strength’. Response options were: (i) yes; (ii) no; (iii) no, but we would like to; and (iv) don’t know.

### (b) Which methods of representing probability do participants prefer?

As can be seen in table 2, we find that the map, fan chart and error bar received the highest preference ratings, while the tercile bar graph, spaghetti plot and pie chart received the lowest. It therefore appears that maps and representations of spread are more popular among our sample than representations of discrete categories.

**Table 2.** Mean ratings of preference and familiarity for the probability visualizations presented to participants, along with correlations between preference, familiarity and statistical comfort. For distribution bar graph and pie graph, *n*=46; and for map, fan chart, error bar, spaghetti plot and tercile bar graph, *n*=45.

			correlations (Spearman’s *ρ*)		
	preference mean (s.d.)	familiarity mean (s.d.)	preference with familiarity *ρ*	preference with statistical comfort *ρ*	familiarity with statistical comfort *ρ*
map	3.9 (0.7)	3.3 (1.1)	0.60***	−0.22	−0.06
fan chart	3.9 (0.7)	3.4 (1.1)	0.52***	0.24	0.18
error bar	3.7 (0.7)	3.5 (1.1)	0.74***	0.38**	0.34*
bar graph: distribution	3.4 (0.8)	3.0 (1.1)	0.52***	0.26^#^	0.19
pie chart	3.2 (0.9)	2.9 (1.1)	0.71***	−0.21	−0.17
spaghetti plot	3.1 (1.0)	3.0 (1.2)	0.69***	0.02	0.13
bar graph: tercile	3.0 (0.9)	2.5 (1.1)	0.76***	0.08	0.26^#^

Significant at *p≤0.05, **p≤0.01, ***p≤0.001; marginally significant at ^#^*p*≤0.10.

As our sample size precluded counterbalancing, we cannot entirely discount the possibility that the presentation order may have had some effect on participant preference [54,55]. Hence, it is recommended that any follow up with a larger, less specialized, sample employ counterbalancing or a between-groups design.

### (c) To what extent is preference associated with familiarity and statistical comfort?

For all visualizations, preference was strongly associated with familiarity (table 2), indicating that participants tended to prefer those visualization formats with which they were already most familiar. Comfort with statistics meanwhile had a significant positive association with preference for the error bar. Hence, participants with lower levels of statistical comfort may not be as familiar with or interested in such visual representations.

### (d) What type of information about uncertainty in seasonal and interannual forecasts are users currently receiving, and what else would they like to receive?

**Figure 3.**
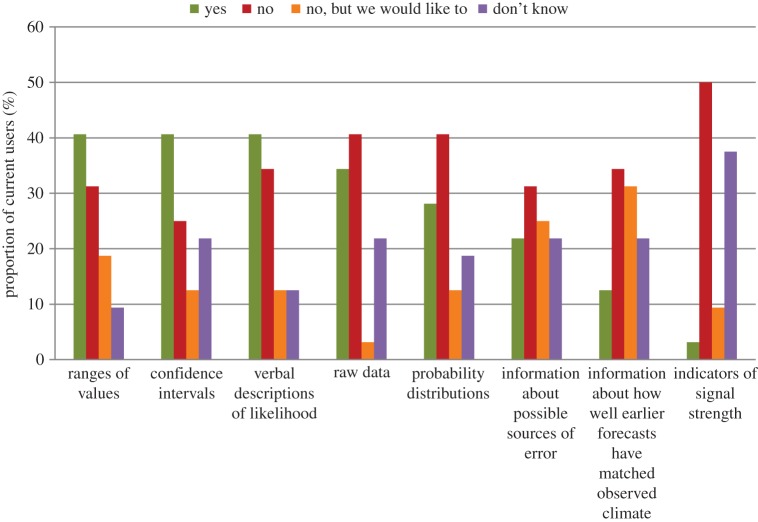
Proportion of current users of seasonal and interannual climate forecast (*n*=32) indicating whether they received different forms of information about uncertainty.

For current users of seasonal or interannual forecasts, figure 3 illustrates the proportion currently receiving the listed forms of information about uncertainty. We find that confidence intervals, ranges of values and verbal descriptions are the most commonly received formats, followed by raw data and probability distributions. Of those who did not currently receive information about raw data only one participant indicated that they would like to receive it. Most interesting, however, is that most users reported that they did not currently receive information about how well forecasts have matched observations (of past climate); although, of those reporting that they do not receive this information, nearly half indicated that they would like to. This suggests that information about the reliability and skill of forecasts is not readily available to many users or that it is presented in a way that is not salient to them. As this information is necessary for users to understand how well forecasts perform relative to climatology (historical averages), this is an area of concern.

## 7. Discussion

Future developments in the quality of seasonal and interannual climate forecasts for Europe may lead to more widespread use in organizational decision-making across various climate-sensitive sectors. In order for these decisions to be appropriately informed, it will be necessary for providers to ensure that both the probabilistic nature of the prediction and its associated skill are adequately communicated. However, not all users will share providers’ expertise in interpreting complex statistical information. Indeed, should use become more widespread it is to be expected that the proportion of users without technical or statistical expertise will grow. The behavioural decision-making literature demonstrates that when it comes to information about uncertainty, there is often a mismatch between the understanding of experts and non-experts. Even where users have technical or statistical expertise mismatches may exist between information provision and user needs. Ambiguity aversion may also mean that some decision-makers are inclined to disregard information where uncertainty is explicitly presented. Through undertaking a survey with a small sample of participants interested in seasonal and interannual climate forecasts, we explored (i) organizational approach to uncertainty, (ii) perceptions of understandability and usefulness, and (iii) preferences for receiving uncertain information.

Overall, reported tolerance for uncertainty and false alarms was relatively high among our sample. It was also evident that while a majority of participants indicated that their organization tended to focus on ‘most likely’ events, this did not preclude concern with low-probability high-impact events. With respect to the processing of uncertain information, we found that while many participating organizations had the capacity to do their own risk analysis most still require providers of climate information to perform some form of data interpretation. Indeed, a majority indicated that their organization liked to receive information in a way that facilitated yes/no decisions. As our participants were characterized by their interest in climate forecasts, it is possible if not likely that this need for interpretation by providers would be even more pronounced in a wider sample. The large proportion of participants indicating that their organization preferred information formats that facilitate yes/no decision-making is in line with findings that a preference for cues that can be used to signal if/then responses has been observed among both expert [40] and non-expert [56,57] user groups. Indeed, where a large amount of decision information and competing demands exist, reliance on simple cues—rather than seeking to process all available information—is in keeping with the principles of bounded rationality [58]. However, the fact that preference for this format was associated with lower tolerance for uncertainty is potentially concerning. While decision aids that take into account users’ tolerance for false alarms can be developed to provide recommendations for action based on climate forecasts [50], if these tools reduce the salience of forecast uncertainty, then they may create a false sense of certainty; potentially leading to maladaptive decision-making [8,9] and a loss of trust in providers [9,10]. Indeed, one recent experimental study has found that decision aids providing explicit information about the likelihood of false alarms can increase trust and elicit better decision-making relative to formats that simply provide recommendations for action [9]. Hence, when creating decision aids incorporating information from climate forecasts, providers must test their materials to ensure that the existence of uncertainty is not obscured [18].

In terms of current users’ perceptions of understandability and usefulness, our results show that there is a mismatch between the perceived usefulness of seasonal and interannual forecasts, and how easy to understand current users find them; with seasonal and interannual forecasts being perceived as more useful than they are understandable. We also find that seasonal and interannual forecasts are considered to be less understandable than weather forecasts. This may of course be explained by the greater familiarity of weather forecasts. Research examining information preferences for hurricane path forecasts [36] and long-term climate change projections [35] has found a tendency for people to prefer familiar formats to novel ones. Likewise, the fact that many weather forecasts are deterministic rather than probabilistic may render them easier to interpret. It is also possible that thinking about the next few days to weeks is easier than the next few months to years. Nonetheless, the disparity in perceived understandability between weather forecasts and seasonal and interannual forecasts suggests that the user-friendliness of forecasts tends to diminish with increasing forecast lead time. It should be kept in mind that participants rated only those forecasts that their organization currently received. We may therefore infer that those who responded to questions about seasonal and interannual forecasts were already motivated to engage with this information. Hence, the low ratings of understandability by this group suggest that perceived difficulty could be a considerable barrier to use among potential users who are not yet as engaged. Our findings therefore highlight a pressing need for more user-friendly formats for presenting climate information at time scales of more than one month in the future.

With respect to preferences for receiving information about uncertainty, we found that maps and representations that show the distribution or ‘spread’ of the forecast (e.g. fan chart, error bars) were preferred to those representing discrete categories (e.g. pie chart, tercile bar graph). These preferences were related to the familiarity of these visualizations; with preference for certain representations of spread being strongest among those reporting greater comfort with statistics. As our sample contained a large proportion of participants with high technical and statistical expertise, it is therefore possible that these formats would be favoured less among a broader sample. Because familiarity informs people’s preferences for information formats [35,36], users may be averse to more novel, less familiar formats in this context. Our findings also indicate that information about forecast reliability and skill is not currently being presented to all users who wish to receive it; or at least not being presented in a way that is well understood by these users. As this information on second-order uncertainty is necessary for forecasts to be used in a fully informed way, this highlights an urgent need for more understandable, user-friendly ways of presenting this information.

It should of course be kept in mind that our survey examined preferences and perceptions as opposed to objective understanding and appropriate use; and that preference [37], familiarity [38,39] and perceived ease of understanding [37] may not always correspond with how well information is actually understood. Indeed, there is some evidence to suggest that users’ confidence in their interpretation of climate information may be associated with the extent to which information is perceived to conform to prior expectations [35]. Hence, further research is needed to examine the extent to which those formats that users prefer are those that are best understood; and, if not, how this disparity can be addressed in a way that does not reduce willingness to use this information. Work will also be needed to establish how skill can be integrated in forecast information in a way that can be understood by users varying in expertise. By addressing these challenges, we may start to close the gap between perceived usefulness and understanding.

## Supplementary Material

User Needs Survey

## Supplementary Material

Visualisations presented to participants

## Supplementary Material

Sectoral breakdown of participants

## Supplementary Material

Survey data

## Data accessibility

A full list of the survey questions presented to participants along with the anonymized response data underpinning our statistical analyses has been uploaded as part of the electronic supplementary material.
