# Supplementary material for: Communicating uncertainty in seasonal and interannual climate forecasts in Europe
Source: Philos Trans A Math Phys Eng Sci. 2015 Nov 28;373(2055):20140454. doi: 10.1098/rsta.2014.0454 (PMC4608030; doi:10.1098/rsta.2014.0454)
Supplement: User Needs Survey [file rsta20140454supp1.doc]

**Sectoral breakdown of participants**

Number of participants per sector

|  | | | | |
| --- | --- | --- | --- | --- |
|  | Survey started (n=50) | | Completed survey(n=44) | |
| Sector | n | % | n | % |
| Water | 10 | 20 | 9 | 20 |
| Energy | 7 | 14 | 6 | 14 |
| Health | 6 | 12 | 6 | 14 |
| Forestry | 6 | 12 | 6 | 14 |
| Tourism | 4 | 8 | 4 | 9 |
| Multi-sectoral | 4 | 8 | 2 | 5 |
| Agriculture | 4 | 8 | 3 | 7 |
| Climate consultancy | 3 | 6 | 3 | 7 |
| Roads | 2 | 4 | 1 | 2 |
| Food Security | 1 | 2 | 1 | 2 |
| Finance | 1 | 2 | 1 | 2 |
| Environment | 1 | 2 | 1 | 2 |
| Emergency planning | 1 | 2 | 1 | 2 |

**Supplementary Table 1:** Number of participants per sector
