# Supplementary material for: Communicating uncertainty in seasonal and interannual climate forecasts in Europe
Source: Philos Trans A Math Phys Eng Sci. 2015 Nov 28;373(2055):20140454. doi: 10.1098/rsta.2014.0454 (PMC4608030; doi:10.1098/rsta.2014.0454)
Supplement: Visualisations presented to participants [file rsta20140454supp2.doc]

**Visualisations presented to participants**

Full size reproductions of the visualisations presented to participants in our user needs survey. Supplementary Figures 1-6 were provided for use in the survey by Mike Butts (DHI) and represent a hypothetical streamflow forecast. Summplmentary Figure 7 was provided for use in the survey by Christoph Spirig (MeteoSwiss) and represents a hypothetical temperature forecast.

Bar Graph


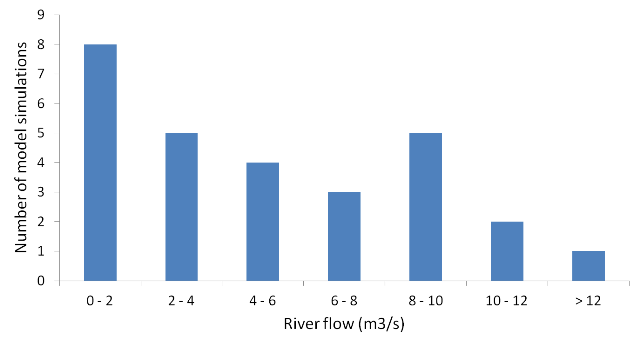


**Supplementary Figure 1:** A frequency graph of the number of model simulations (out of 28) that predict that river flow will be within specific ranges of m3/s (cubic metres per second) over the next 12 months.

Pie graph


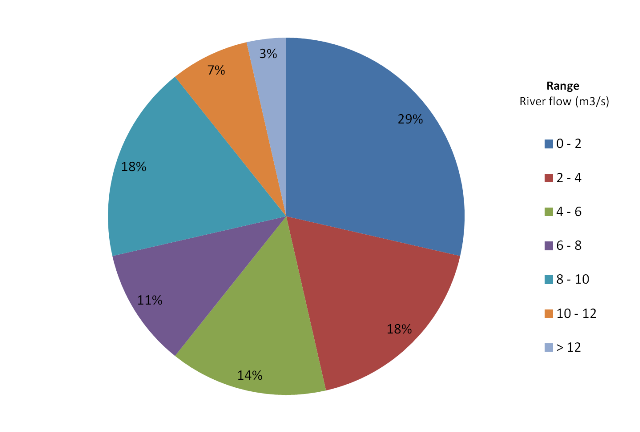


**Supplementary Figure 2** A pie graph showing the proportion of simulations (out of 28) that predict that average river flow will be within specific ranges of m3/s (cubic metres per second) over the next 12 months.

Error bars


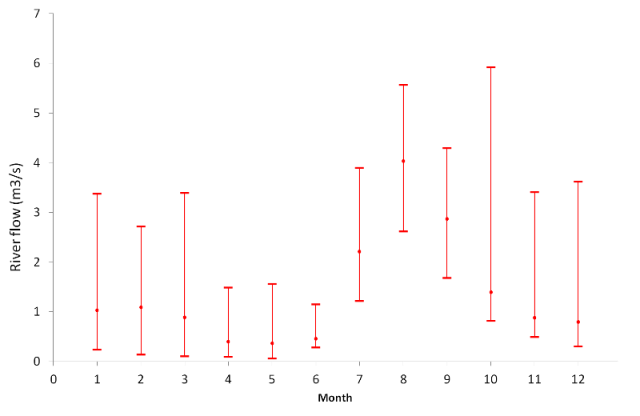


**Supplementary Figure 3:** Error bars showing average predicted river flow by month for twelve months. These are based on 28 daily model simulations. The upper and lower limits of the line represent the minimum and maximum value generated by the simulations. The dot represents the mean.

Fan graph


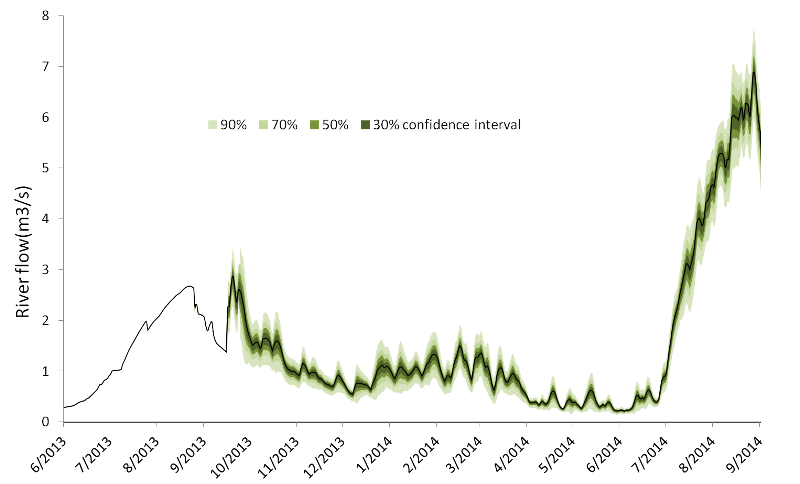


**Supplementary Figure 4:** A fan graph showing predicted change in river flow over time. The thin black line at the start represents recent observations (i.e. actual river flow in the recent past). The coloured areas represent confidence levels around the mean. These confidence levels are based on 28 daily model simulations.

Spaghetti graph


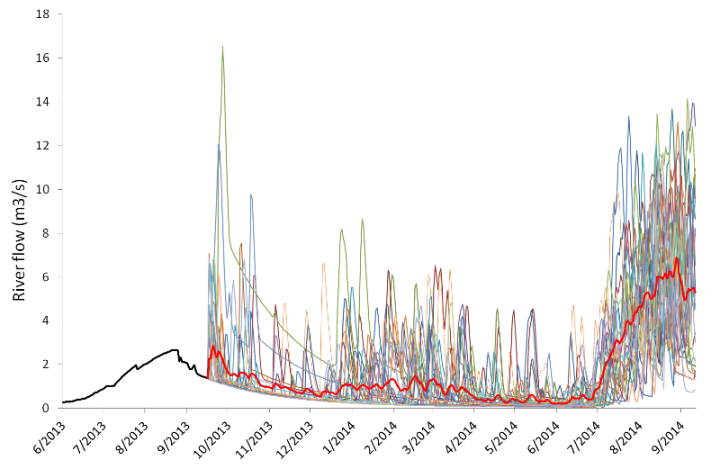


**Supplementary Figure 5:** A spaghetti graph showing predicted change in river flow over time. The black line at the start represents recent observations (i.e. actual river flow in the recent past), the thin coloured lines represent 28 daily model simulations and the thick red line represents the mean of the simulations.

Tercile bar


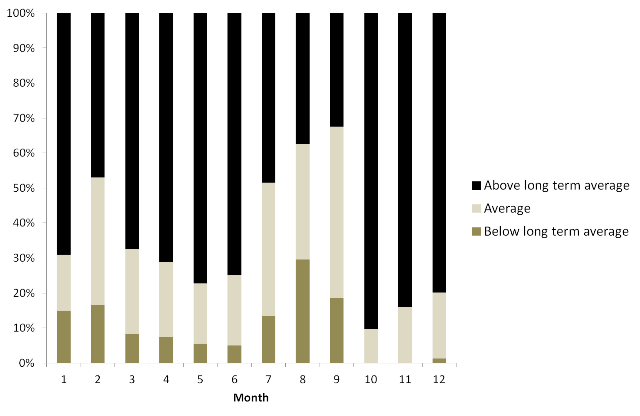


**Supplementary Figure 6:**  Graph indicating the likelihood that river flow will be above the long term average, around the long term average, or below the long term average for each month.

Map


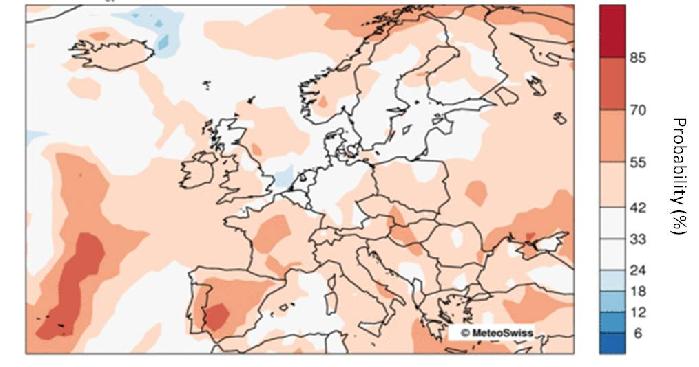


**Supplementary Figure 7:** A map showing the predicted likelihood that average temperature over a three month period will be greater than the long term average.
