# Supplementary material for: Communicating uncertainty in seasonal and interannual climate forecasts in Europe
Source: Philos Trans A Math Phys Eng Sci. 2015 Nov 28;373(2055):20140454. doi: 10.1098/rsta.2014.0454 (PMC4608030; doi:10.1098/rsta.2014.0454)
Supplement: Sectoral breakdown of participants [file rsta20140454supp3.doc]

**User Needs Survey**

A full list of all questions presented to participants in our user needs survey

**Section 1**

Q1. Is your organisation involved in the EUPORIAS project?

Yes No

Don't Know

Q2 Name (Optional)

Q3 Organisation (Optional)

Q4 Email (Optional)

Q5 What is your role within your organisation?

Q6 Which sector(s) does your organisation belong to?

Water

Energy

Health

Forestry

Tourism

Agriculture

Insurance/finance

Emergency planning

Other (please provide details) ____________________

Q7 Does your organisation make use of any of the following types of weather and climate information?

Forecasts for up to four weeks in the future

Forecasts for between one month and one year in the future

Forecasts for between one year and ten years in the future

Forecasts for a decade or more in the future

Responses given on a scale of a scale of:

Yes

No

Don't Know

Q8 Are there any other types of forecast or projection about the future that are important to your organisation (e.g. economic growth forecasts, consumer demand forecasts)? (List up to 3)

1____________________

2____________________

3____________________

Q9 On a scale of 1 to 5 how easy to find (or access) do you think the following are? [NOTE: ONLY THOSE ITEMS RESPONDENTS HAD PREVIOUSLY INDICATED THAT THEY USED WERE DISPLAYED – IF RESPONDENTS INDICATED THAT THEY DIDN’T USE ANY THEY AUTOMATICALLY FORWARDED TO SECTION 2]

Forecasts for up to four weeks in the future

Forecasts for between one month and one year in the future

Forecasts for between one year and ten years in the future

Forecasts for a decade or more in the future

Respondent listed forecast 1

Respondent listed forecast 2

Respondent listed forecast 3

Responses given on a scale of a scale of:

1 Not at all easy to find

2

3

4

5 Very easy to find

Don’t Know

Q9.a Are there any comments about this question that you would like to make? (Optional)

Q10 On a scale of 1 to 5, how easy to understand do you think the following are?

Forecasts for up to four weeks in the future

Forecasts for between one month and one year in the future

Forecasts for between one year and ten years in the future

Forecasts for a decade or more in the future

Respondent listed forecast 1

Respondent listed forecast 2

Respondent listed forecast 3

Responses given on a scale of a scale of:

1 Not at all easy to understand

2

3

4

5 Very easy to understand

Don’t Know

Q10.a Are there any comments about this question that you would like to make? (Optional)

Q11 On a scale of 1 to 5, how useful do you think the following are?

Forecasts for up to four weeks in the future

Forecasts for between one month and one year in the future

Forecasts for between one year and ten years in the future

Forecasts for a decade or more in the future

Respondent listed forecast 1

Respondent listed forecast 2

Respondent listed forecast 3

Responses given on a scale of a scale of:

1 Not at all useful

2

3

4

5 Very useful

Don’t Know

Q11.a Are there any comments about this question that you would like to make? (Optional)

**Section 2**

Q12 Thinking about your organisation's approach to dealing with confidence and uncertainty in general, please rate your level of agreement with the statements below.

“We plan for rare but severe events."

“My organisation has clear guidelines as to how much statistical confidence is required before we can take action.”

"When it comes to risk management, we mainly focus on those risks that are most likely to occur."

"We do our own risk modelling."

Responses given on a scale of a scale of:

1 Strongly disagree

2 Disagree

3 Neither agree nor disagree

4 Agree

5 Strongly agree

Don’t Know/Not applicable

Q12.a Are there any comments about this question that you would like to make? (Optional)

Q13 Thinking about your organisation's approach to dealing with confidence and uncertainty in general, please rate your level of agreement with the statements below.

"Time pressure means that we sometimes have to make decisions before we have as much information as we would like."

“We need to know what will happen, not what might happen.”

"We don’t tend to focus on events that have a very low chance of occurring.”

"We like to receive information in a form that helps us to make Yes/No decisions."

Responses given on a scale of a scale of:

1 Strongly disagree

2 Disagree

3 Neither agree nor disagree

4 Agree

5 Strongly agree

Don’t Know/Not applicable

Q13.a Are there any comments about this question that you would like to make? (Optional)

Q14 Thinking about your organisation's use of climate information specifically, please rate your level of agreement with the following statements. If your organisation doesn't presently use any form of weather or climate information select 'Don't know/Not applicable'.

“It is important for us to consider what might happen in a 'worst case scenario' as well as what is most likely to happen"

“When it comes to predicting extreme weather events we are willing to accept more false alarms if it means that a greater number of real extreme events are detected in advance.”

“We really just need raw model data so that we can do our own analysis.”

Responses given on a scale of a scale of:

1 Strongly disagree

2 Disagree

3 Neither agree nor disagree

4 Agree

5 Strongly agree

Don’t Know/Not applicable

Q14.a Are there any comments about this question that you would like to make? (Optional)

**Section 3**

Q15 Which of the following best describes your feelings about working with statistics and numerical information, such as means, percentages, confidence levels, and more advanced forms of analysis?

1. “I am not comfortable using statistics or numerical information”
2. “I am comfortable using basic statistics and numerical information” (e.g. means, percentages, frequency counts)
3. “I am comfortable using more complex statistics and numerical information” (e.g. confidence levels, probability distributions)
4. “I am comfortable using standard statistical tests” (e.g. correlations, t-tests)
5. “I am comfortable using more advanced statistical techniques” (e.g. Monte Carlo simulations, mathematical modelling)
6. Other (Please give details) ____________________

Q16 Do you use statistics or numerical information in your day to day work?

Yes

No

Q16.a What kind of statistics or numerical information do you use? (Select all that apply) [PRESENTED ONLY IF RESPONDENT SELECTED YES TO Q16]

Frequency counts

Percentages

Measures of averages (e.g. mean, median, mode)

Exceedance thresholds (e.g. as might be represented by return periods)

Measures of spread (e.g. variance, standard deviation, confidence levels)

Probability distributions

Other (please provide details) ____________________

Q17 Below are three different ways of describing the likelihood of it raining tomorrow. Which of these do you prefer? (Required)

There is a 30% chance of rain tomorrow

There is a .3 chance of rain tomorrow

There is a 3 in 10 chance of rain tomorrow

I would prefer another format (please provide details)

____________________

Q18 – 23

[INSTRUCTIONS]

On the next few screens you will be presented with graphs representing uncertainty in a hypothetical river flow forecast. All graphs are based on the same underlying data. We would like you to look at them and rate what you think of each type of graph on the scales provided.

You and your organisation may not be interested in river flow itself, but these types of graphs can also be used to show information about confidence and uncertainty in forecasts for other climate and climate impact variables such as temperature and rainfall. Therefore, we ask that you base your ratings on your thoughts about the style of the graphs rather than whether you would be interested in using information about river flow itself.

Note: some images may take a few seconds to load. When you are ready to see the graphs click "Next"

[Respondents were presented with a series six hypothetical streamflow forecast visualisations (see Appendix II for illustrations)]

Bar graph

Pie graph

Error bar

Fan graph

Spaghetti graph

Tercile bar

[Respondents rated their opinion of each on a set of six scales]

"This type of graph is useful"

"I would use this type of graph in my decision making"

"I would share this type graph with a colleague, for them to use in their own decision making."

"This graph is easy to understand"

"I like this graph."

"I use graphs like this in my work."

Responses given on a scale of a scale of:

1 Strongly disagree

2 Disagree

3 Neither agree nor disagree

4 Agree

5 Strongly agree

Q18.a – 23.a Are there any comments about this type of graph that you would like to make? (Optional)

Q19 On the next screen you will be shown a map representing a hypothetical seasonal temperature forecast for Europe (please note this does not represent a current forecast). We would like you to look at the map and rate what you think on the scales provided.

Temperature might not interest your organisation, but this style of map can be used to present information about a range of different climate and climate impact variables. We therefore ask that you base your ratings on what you think of the type of graph rather than your interest in temperature.

[Respondents rated their opinion of the map (see Appendix II) on a set of six scales]

"This type of map is useful"

"I would use this type of map in my decision making"

"I would share this type of map with a colleague, for them to use in their own decision making."

"This map is easy to understand"

"I like this map."

"I use maps like this in my work."

Responses given on a scale of a scale of:

1 Strongly disagree

2 Disagree

3 Neither agree nor disagree

4 Agree

5 Strongly agree

Q19.a Are there any comments about this type of map that you would like to make? (Optional)

Q20. Below is a list of ways in which information about probabilities and quantities can be shown. Have you ever used them in your work? (Please select Yes if you have and No if you have not)

Numeric tables

Bar graphs

Pie graphs

Histograms

Maps

Error bars

Probability density functions

Cumulative probability density functions

Spaghetti graphs

Other (please provide details) ____________________

Responses given as:

Yes

No

Q21 If you were completely free to choose how you received information about uncertainty in the climate or climate impacts your organisation is interested in what would you choose? (Optional)

**Section 4 [CURRENT USERS OF SEASONAL OR DECADAL INFORMATION ONLY]**

Q22. Your answers to previous questions indicate that you currently use climate forecasts at a seasonal to decadal timescale.That is to say climate forecasts for between one month and ten years in the future. Which climate variables and impacts does your organisation get these forecasts for (tick all that apply)

Temperature (land)

Rainfall

Cloud cover

Wind

Temperature (sea)

River flow

Crop yields

Extreme indices (e.g. heat, cold)

Other (please provide details) ____________________

Q23 How often does your organisation use climate forecasts for the following timescales?

1 - 3 months in the future

4 - 6 months in the future

7 -12 months in the future

1 - 2 years in the future

3 - 5 years in the future

6 - 10 years in the future

Responses given on a scale of:

1 Never

2 Not often

3 Often

4 Very often

Don’t know

Q24Does your organisation get any of the following types of information about uncertainty in its seasonal to decadal climate forecasts? (Please note: the things listed here may not be available for all types of variables and indices)

Ranges of values (e.g. 'temperature projected to be between 18C and 23C')

Confidence levels

Probability distributions

Indicators of signal strength

Raw data

Verbal descriptions of likelihoods (e.g. descriptions such as 'likely' or 'unlikely')

Information about possible sources of error in the models used

Information about how well earlier forecasts have matched observed climate variables and impacts.

Responses given on a scale of:

1 Yes

2 No

3 Often

4 No, but we would like to

Don’t know

Q25 Do you receive information about uncertainties in any other form?

Yes (Please provide details) (1) ____________________

No (2)

Q26 Are there any comments about any of these questions that you would like to make? (Optional) [REFERRING TO Q22 - Q25]

Q27 On the last screen you indicated that you get the following types of information about uncertainty in climate forecasts. How often would you say that you use this information in your decision making? [NOTE: ONLY ITEMS RESPONDENTS INDICATED THAT THEY RECEIVED WERE DISPLAYED – IF RESPONDENTS DIDN’T RECEIVE ANY OF THE LISTED FORMS OF INFORMATION THEY DID NOT SEE THIS QUESTION]

Ranges of values (e.g. 'temperature projected to be between 18C and 23C')

Confidence levels

Probability distributions

Indicators of signal strength

Raw data

Verbal descriptions of likelihoods (e.g. descriptions such as 'likely' or 'unlikely')

Information about possible sources of error in the models used

Information about how well earlier forecasts have matched observed climate variables and impacts.

Responses given on a scale of:

1 Never

2 Not often

3 Often

4 Very often

Don’t know

Q28 Do you feel that the information you currently receive regarding climate forecasts at a seasonal to decadal timescale fully meets your needs?

Responses given as:

Yes

No

Q28.a How could this be changed so that your needs are met? [IF “NO” SELECTED FOR Q28]

**Section 4 [NON-USERS OF SEASONAL OR DECADAL INFORMATION ONLY]**

Q30 Why don't you use currently forecasts at a seasonal to decadal timescale? (tick all that apply)

The matter has never been discussed in our organisation

There is too much uncertainty in these forecasts for them to be useful in our decision making

The information available isn't precise enough for us to use in our decision making

No forecasts are available for the events or indices that we are interested

We have not been able to find a suitable provider

The information is not provided in a way that we can use

Other (please provide details) ____________________

Q30a How precise would seasonal to decadal forecasts have to be to be useful to you? [IF RESPONDENT CHECKED “THE INFORMATION AVAILABLE ISN'T PRECISE ENOUGH FOR US TO USE IN OUR DECISION MAKING”]

Q30b Why isn't the information usable? (tick all that apply) [IF RESPONDENT CHECKED “THE INFORMATION IS NOT PROVIDED IN A WAY THAT WE CAN USE”]

The information available is not provided in a way that is easy to understand

The information available does not contain enough detail about uncertainty

The information available does not contain enough detail in general

Other (please provide details) ____________________

Q31Are there any comments about any of these questions that you would like to make? (Optional) [REFERRING TO Q29 - Q30]

**Section 4 [NON-USERS OF SEASONAL OR DECADAL INFORMATION ONLY]**

Q30 Why don't you use currently forecasts at a seasonal to decadal timescale? (tick all that apply)

The matter has never been discussed in our organisation

There is too much uncertainty in these forecasts for them to be useful in our decision making

The information available isn't precise enough for us to use in our decision making

No forecasts are available for the events or indices that we are interested

We have not been able to find a suitable provider

The information is not provided in a way that we can use

Other (please provide details) ____________________

Q30a How precise would seasonal to decadal forecasts have to be to be useful to you? [IF RESPONDENT CHECKED “THE INFORMATION AVAILABLE ISN'T PRECISE ENOUGH FOR US TO USE IN OUR DECISION MAKING”]

Q30b Why isn't the information usable? (tick all that apply) [IF RESPONDENT CHECKED “THE INFORMATION IS NOT PROVIDED IN A WAY THAT WE CAN USE”]

The information available is not provided in a way that is easy to understand

The information available does not contain enough detail about uncertainty

The information available does not contain enough detail in general

Other (please provide details) ____________________

Q31Are there any comments about any of these questions that you would like to make? (Optional) [REFERRING TO Q29 - Q30]

**Section 5 [OPTIONAL]**

Q32 Gender (Optional)

Male

Female

Q33 Age (Optional)

Q34 How long have you worked in your current field? (Optional)

Q35 What is your highest level of formal education? (Optional)

High School

Undergraduate degree

Postgraduate degree

Other (please provide details) ____________________

Q35.a What was your degree subject? (If you have more than one degree list all of them) (Optional) [DISPLAYED ONLY IF RESPONDENT SELECTED UNDERGRADUATE DEGREE OR POSTGRADUATE DEGREE FOR Q35]

Q29 Your answers to previous questions indicate that you do not currently use climate forecasts at a seasonal to decadal timescale. That is to say climate forecasts for between one month and ten years in the future.  If you were to receive forecasts at this timescale, which climate and climate impact variables would you be interested in receiving forecasts for? (tick all that apply)

Temperature (land)

Rainfall

Cloud cover

Wind

Temperature (sea)

River flow

Crop yields

Extreme indices (e.g. heat, cold)

Other (Please provide details) ____________________
